# Modulation of Aβ _42_-induced toxic effects on the cultured neuronal network activity by extracellular matrix stiffness: The toxicity of Aβ to nerves depends on the stiffness of the extracellular matrix

**DOI:** 10.3724/abbs.2025095

**Published:** 2025-07-09

**Authors:** Zhongliang Wei, Hucheng Zhao, Chandramohan Muruganandham, Chongdong Jian

**Affiliations:** 1 The Affiliated Hospital of Youjiang Medical University for Nationalities Baise 533000 China; 2 Faculty of Pharmacy and Biomedical Sciences MAHSA University SP2 Bandar Sauiana Putra Selangor 42610 Malaysia; 3 Institute of Biomechanics and Medical Engineering Department of Engineering Mechanics Tsinghua University Beijing 100084 China

Alzheimer’s disease (AD) is the most common neurodegenerative disease that usually begins with short-term memory loss, gradually progresses to cognitive dysfunction and causes loss of body function and eventual death [1]. Mutations in the *APP3* gene encoding the Aβ precursor protein (APP) are known to cause early-onset AD and suggest that Aβ is a major factor in AD development [2]. Enzyme complexes, such as α-, β- and γ-secretases, catalyze various cleavage pathways to produce a variety of Aβ isoforms of different lengths [3]. These Aβ peptides have proven toxic to the brain and accumulate in AD to form cerebral plaques. The main isoform of Aβ present in these plaques is the 42 amino acid variant known as Aβ _42_ [4]. A previous study revealed that changes in the stiffness of the extracellular matrix (ECM) can induce remodeling of the cytoskeleton of neurons in the brain tissues of AD patients, leading to changes in the morphology and function of neurons [5]. ECM stiffness is unique to each specific tissue, and resident cells have developed to function optimally in microenvironments with specific ECMs [6]. Brain tissues are reported to have a Young’s modulus of elasticity between 0.1 and 16 kPa. In patients with AD, a decrease in the elasticity of brain tissues was detected [7]. Interestingly, the ECM is known to play an important role in cytoskeleton remodeling and neuronal function [8], and a stiff ECM has been reported to promote actin polymerization and stress fiber formation, whereas a soft ECM triggers actin depolymerization [9]. However, it remains uncertain whether alterations in ECM stiffness in the AD brain contribute to Aβ-induced toxicity, particularly considering that Aβ is recognized to cause neuronal toxicity by disrupting the actin cytoskeleton, which leads to subsequent synaptic and dendritic abnormities [10]. As such, the present study aimed to investigate the effects of substrate stiffness on Aβ-induced toxicity to the neuronal network in cultured neurons.

Hippocampal neurons cultured on soft and stiff substrates were assessed for cell viability by MTT assay. When the neuronal cultures were exposed to 1 μM Aβ _42_ for 48 h, there was a significant decrease in the viability of the cells cultured on the stiff substrates ( Supplementary Figure S1), but there was no significant effect on the viability of the neuronal cultured on the soft substrates ( Supplementary Figure S1). In addition, the influence of Aβ _42_ on the number of synapses within the cultured neuronal network was analyzed using confocal immunofluorescence imaging. This analysis revealed that Aβ _42_ exposure induced a decrease in synaptic formation in cultured neurons, which was dependent on substrate stiffness ( Supplementary Figure S2).

To evaluate the effect of substrate stiffness on Aβ _42_-induced toxicity to synaptic transmission in the cultured neuronal network, spontaneous Ca ^2+^ oscillations were examined in neurons cultured on substrates with different stiffness treated with Aβ _42_. The percentage of neurons with spontaneous Ca ^2+^ oscillations was significantly greater in neurons cultured on stiff substrates than in those cultured on soft substrates ( Figure 1A,B). After Aβ _42_ exposure, the percentage of neurons with spontaneous Ca ^2+^ oscillations was significantly decreased in neurons cultured on the stiff substrates ( Figure 1C). Exposure to Aβ _42_ only slightly influenced the percentage of spontaneous Ca ^2+^ oscillations in neurons cultured on the soft substrate ( Figure 1C). The amplitude and frequency of spontaneous Ca ^2+^ oscillations were significantly greater in neurons cultured on the stiff substrates than in those cultured on the soft substrates ( Figure 1D,E). After exposure to Aβ _42_, the amplitude and frequency of spontaneous Ca ^2+^ oscillations were significantly reduced in neurons cultured on stiff substrates ( Figure 1D,E). However, exposure to Aβ _42_ had only a weak influence on the amplitude and frequency of spontaneous Ca ^2+^ oscillations in neurons cultured on soft substrates ( Figure 1D,E). To further investigate the effects of substrate stiffness on synapse function following exposure to Aβ _42_, spontaneous postsynaptic currents were recorded in DIV14-16 neurons cultured on stiff and soft substrates. The percentage of neurons with spontaneous postsynaptic currents was considerably greater in neurons cultured on the stiff substrates than in those cultured on the soft substrates ( Figure 2A,B). Following exposure to Aβ _42_, the percentage of neurons with spontaneous postsynaptic currents on the stiff substrate significantly decreased ( Figure 2C). In contrast, exposure to Aβ _42_ had no significant effect on neurons with spontaneous postsynaptic currents on the soft substrate ( Figure 2C). The amplitude and frequency of sEPSCs and sIPSCs were significantly greater in neurons cultured on stiff substrates than in those cultured on soft substrates ( Figure 2D,E). Following exposure to Aβ _42_, the amplitude and frequency of sEPSCs and sIPSCs were significantly decreased in neurons cultured on the stiff substrates ( Figure 2D,E). However, exposure to Aβ _42_ only slightly affected the amplitude and frequency of spontaneous excitatory postsynaptic currents (sEPSCs) and inhibitory postsynaptic currents (sIPSCs) in neurons cultured on the soft substrate ( Figure 2D,E). The effects of exposure to Aβ _42_ on evoked action potential-evoked postsynaptic currents were also determined. Evoked excitatory transmitter release and excitatory postsynaptic currents are heightened at the synapses of neurons cultured on stiff substrates. Conversely, evoked postsynaptic currents are infrequently observed in neurons cultured on soft substrates. Following exposure to Aβ _42_, the percentage of evoked postsynaptic currents in neurons cultured on the stiff substrate significantly decreased ( Supplementary Figure S3). Collectively, these results indicate that substrate stiffness has a significant effect on Aβ _42_-induced neurotoxicity and that increased stiffness can worsen Aβ _42_-induced toxic effects on neuronal functions.

**Figure 1:**
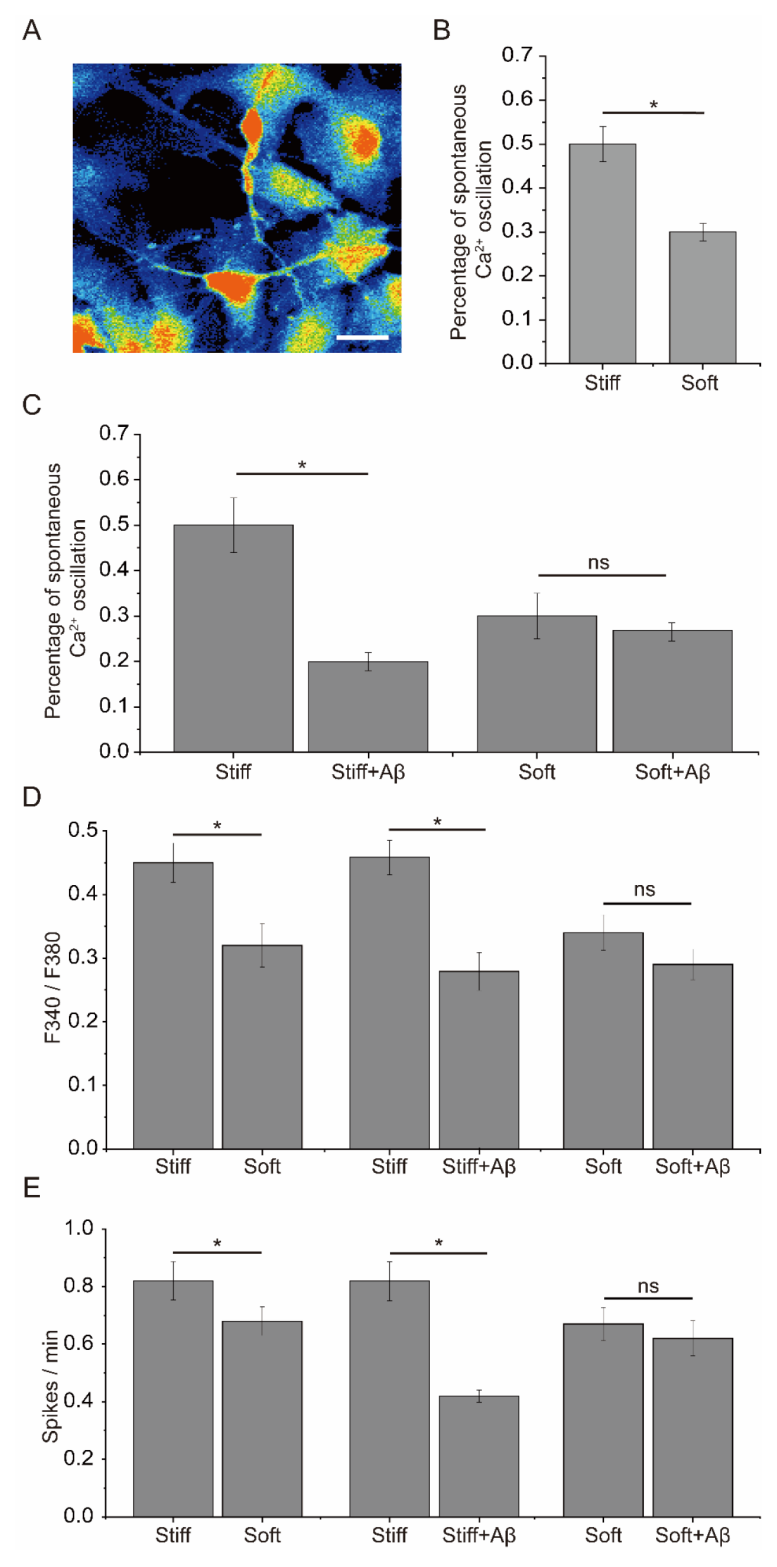
Figure 1 Effect of PA gel substrate stiffness on spontaneous cytosolic Ca ^2+^ oscillations in cultured neuronal networks following exposure to Aβ _42_ (A) Representative single-cell image showing Ca2+ fluorescence in DIV 14-16 hippocampal neurons. (B) The percentage of neurons with Ca2+ oscillations in neurons cultured on stiff and soft substrates (n = 6). (C) The percentage of neurons with Ca2+ oscillations among neurons cultured on stiff and soft substrates with or without exposure to Aβ42 (n = 6). (D) The magnitude of Ca2+ oscillations in 58–62 neurons cultured on soft and stiff substrates with or without exposure to Aβ42 from three preparations. (E) Frequencies of Ca2+ oscillations in 58–62 neurons cultured on soft and stiff substrates with or without exposure to Aβ42 from three preparations. Scale bar: 20 μm. * denotes a significant difference with P < 0.05, and ns denotes no significant difference.

**Figure 2:**
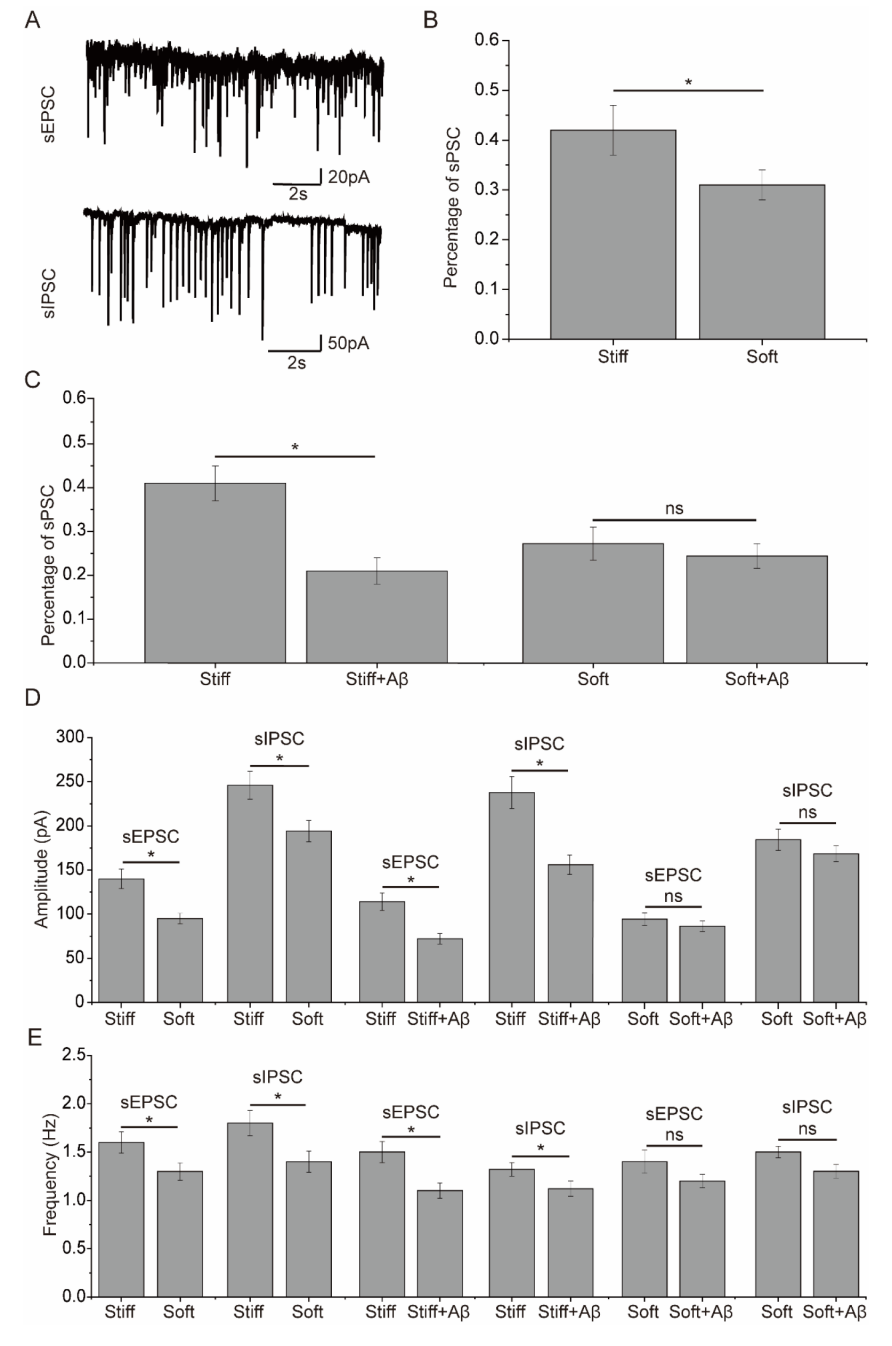
Figure 2 Effect of PA gel substrate stiffness on spontaneous excitatory postsynaptic current (sEPSC) and inhibitory postsynaptic current (sIPSC) in cultured neurons following exposure to Aβ _42_ (A) Representative recordings of sEPSCs and sIPSCs in DIV14-16 neurons. (B) The percentage of neurons with spontaneous postsynaptic currents (sPSCs) on soft and stiff substrates from three preparations (n = 64). (C) The percentage of neurons with sEPSCs and sIPSCs on stiff and soft substrates with or without Aβ42 exposure from three preparations (n = 6–8). (D) The amplitudes of sEPSCs and sIPSCs in 58–62 neurons cultured on soft and stiff substrates with or without exposure to Aβ42 from three preparations. (E) Frequencies of sEPSCs and sIPSCs in 58–62 neurons cultured on soft and stiff substrates with or without exposure to Aβ42 from three preparations. * denotes a significant difference with P < 0.05, and ns denotes no significant difference.

In summary, the main findings of this study suggest that ECM stiffness influences Aβ-induced toxicity to neurons, as Aβ was found to be notably more toxic to neurons cultured on stiff substrates than to those cultured on soft substrates. Overall, the physical signals provided by the ECM can modulate neuronal responses to Aβ toxicity. These findings provide new targets for the development of potential therapeutic interventions for AD.

## Supporting information

25017Supplementary_figures

## Supplementary Data

Supplementary data is available at *Acta Biochimica et Biophysica Sinica* online.
