# Supplementary material for: Modulation of Aβ 42-induced toxic effects on the cultured neuronal network activity by extracellular matrix stiffness : The toxicity of Aβ to nerves depends on the stiffness of the extracellular matrix
Source: Acta Biochim Biophys Sin (Shanghai). 2025 Jul 9;57(11):1904–7. doi: 10.3724/abbs.2025095 (PMC12666660; doi:10.3724/abbs.2025095)
Supplement: 25017Supplementary_figures [file 25017Supplementary_figures.docx]

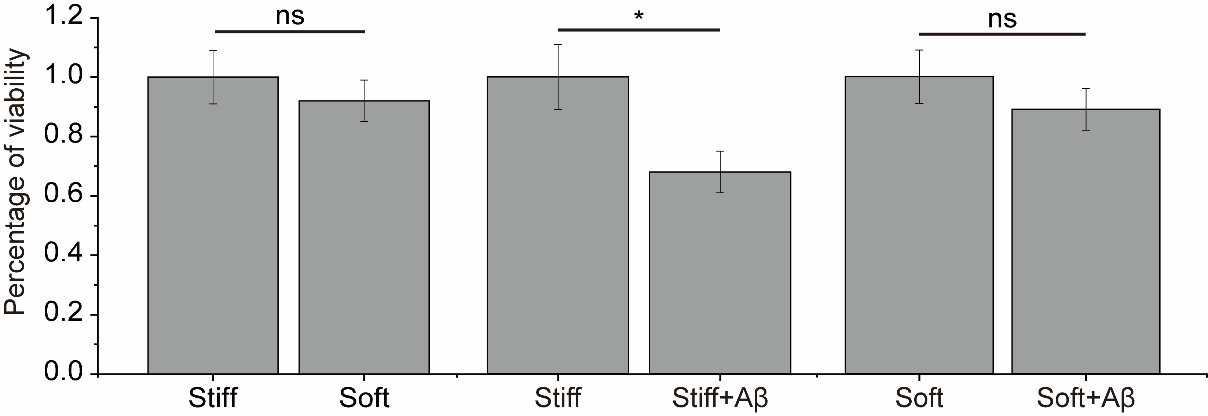


**Supplementary Figure S1. Viability of primary hippocampal neurons cultured on stiff and soft PA gel substrates with different stiffness with or without exposure to Aβ_42_, determined by MTT assay (*n* = 4).** * denotes significant difference with *P* < 0.05, and ns denotes no significant difference.


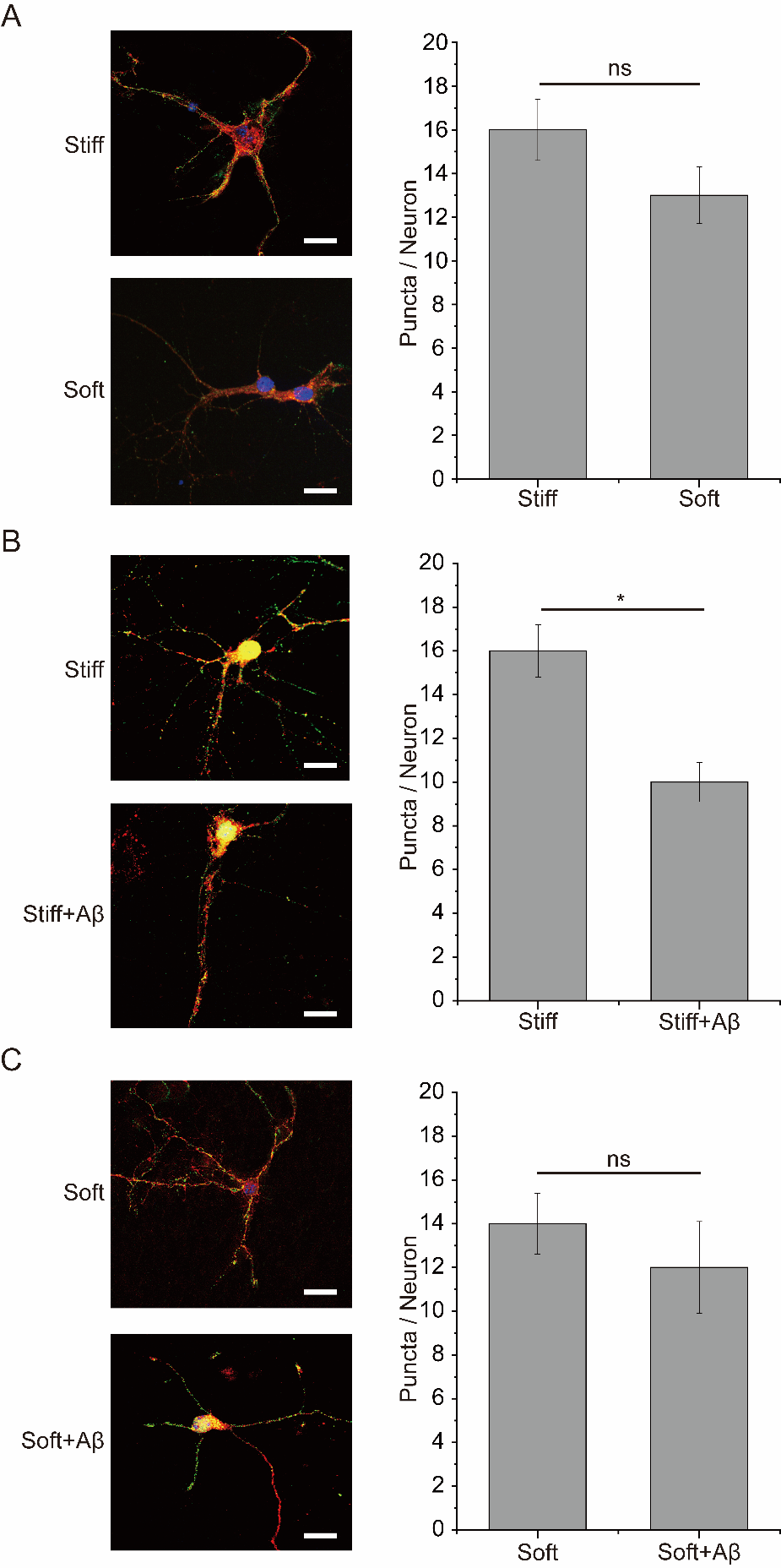


**Supplementary Figure S2. Effect of PA gel substrate stiffness on synapse formation within cultured neuronal networks following Aβ_42_ exposure**  (A) Immunostaining of hippocampal neurons identifying colocalization of presynaptic synaptotagmin (red) and postsynaptic PSD-95 (green) markers in DIV 14−16 neurons cultured on substrates with different stiffness (left panels) and the numbers of synapses (right, *n* = 50). (B) Immunostaining of hippocampal neurons identifying colocalization of presynaptic synaptotagmin (red) and postsynaptic PSD-95 (green) in DIV 14-16 neurons cultured on the stiff substrate with or without Aβ_42_ exposure (left panels) and the numbers of synapses (right, *n* = 50). (C) Immunostaining of hippocampal neurons identifying colocalization of presynaptic synaptotagmin (red) and postsynaptic PSD-95 (green) in DIV 14-16 neurons cultured on the soft substrate with or without Aβ_42_ exposure (left panels) and the numbers of synapses (right, *n* = 50). Scale bar: 20 μm. * denotes significant difference with *P* < 0.05, and ns denotes no significant difference.


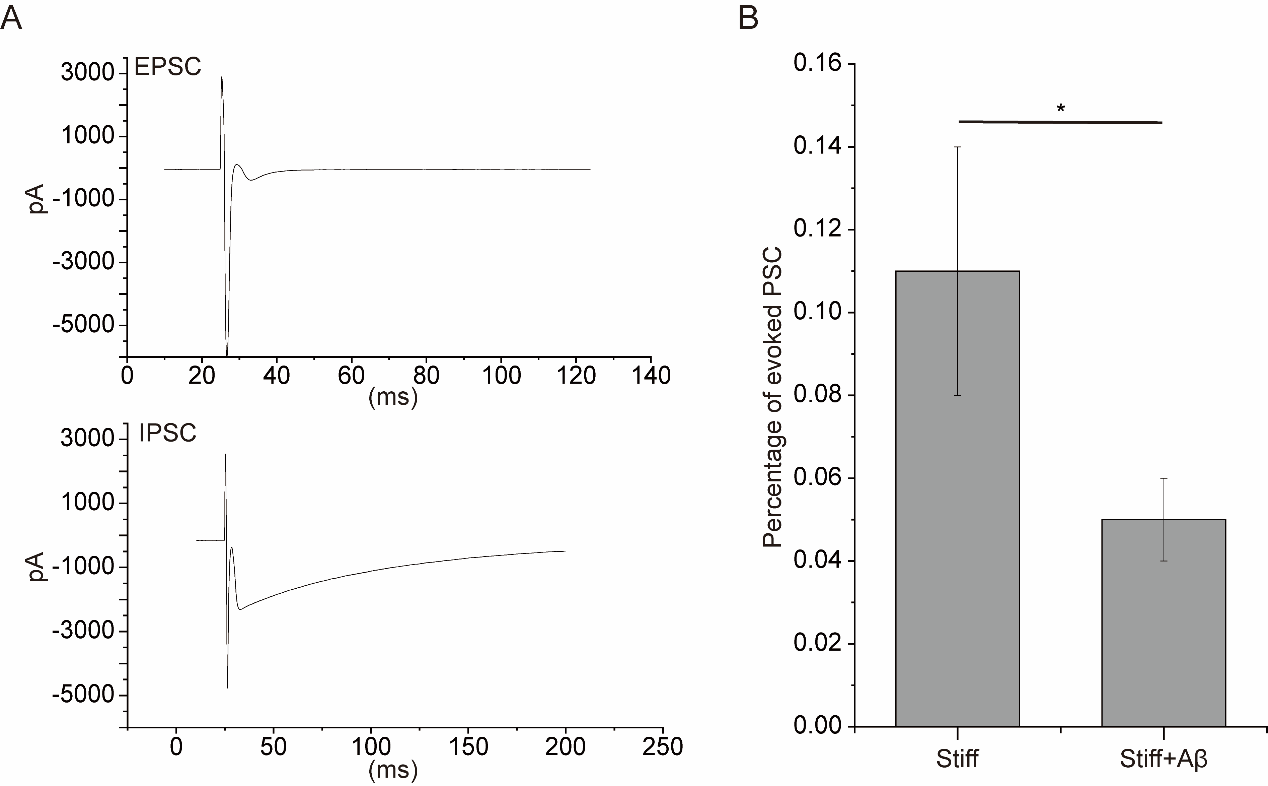


**Supplementary Figure S3. Effect of PA gel substrate stiffness on evoked EPSC and evoked IPSC in cultured neurons following Aβ_42_ exposure**  (A) Representative recordings of evoked EPSC (above) and evoked IPSC (below) in DIV14−16 hippocampal neurons. (B) The percentage of neurons with evoked post-synaptic currents (PSC) on the stiff substrate with or without Aβ_42_ exposure from four preparations (*n* = 64). * denotes significant difference with *P* < 0.05.
